# Distribution of BCR::ABL1 Transcripts in the Different Clinical Phases of Chronic Myeloid Leukemia: Effect on Hematological Parameters and Patient Survival

**DOI:** 10.3390/genes15050567

**Published:** 2024-04-28

**Authors:** Pablo Romero-Morelos, Ana Lilia González-Yebra, Anaid Herrerías-García, Francisco Arath Ruíz-Velázquez, Luis Jonathan Bueno-Rosario, Beatríz González-Yebra

**Affiliations:** 1 Departamento de Investigación, Universidad Estatal del Valle de Ecatepec, Ecatepec 55210, Estado de México, Mexico; 2 Departamento de Ciencias Aplicadas al Trabajo, División Ciencias de la Salud, Universidad de Guanajuato, Campus León, León 37670, Guanajuato, Mexico; 3 Departamento de Medicina y Nutrición, División Ciencias de la Salud, Universidad de Guanajuato, Campus León, León 37670, Guanajuato, México; 4 Unidad de Investigación, Hospital Regional de Alta Especialidad del Bajío, Servicios de Salud del Instituto Mexicano del Seguro Social para el Bienestar (IMSS-BIENESTAR), León 37544, Guanajuato, Mexico

**Keywords:** chronic myeloid leukemia, Philadelphia chromosome, survival, *b3a2*, *b2a2*, *e1a2*

## Abstract

Chronic myeloid leukemia (CML) is a hematopoietic stem cell disorder characterized by the presence of the Philadelphia chromosome, a product of the reciprocal translocation t(9;22)(q34;q11), in the BCR and ABL genes. These rearrangements in both genes lead to the formation of various fusion mRNA products, with preferential expression of *b2a2*, *b3a2*, and other *BCR::ABL1* mRNA variants, combined with additional chromosomal abnormalities. Notably, the distribution and frequency of different mRNA variants vary in different populations. However, studies concerning this in Mexico are limited, and the results have been inconclusive. This study therefore aimed to determine the distribution of *BCR::ABL1* mRNA variants in different clinical phases of CML and their effect on hematological parameters and patient survival. This study included 33 patients, whose demographic, clinical, and molecular data on *BCR::ABL1* mRNA variants and hematological parameters were collected to identify potential associations. A total of 84.8% (n = 28) of patients had *BCR::ABL1* translocation and increased platelet and basophil counts. The most frequent mRNA variant was *b3a2* (64.3%), followed by *b2a2* (28.6%) and *e1a2* (3.6%). Concerning the clinical phases of CML, 75.8% (n = 25), 21.2% (n = 7), and 3% (n = 1) of patients were in the chronic, blast, and accelerated phases, respectively. Moreover, the *b3a2* mRNA variant was more commonly identified in patients in the chronic phase. No correlation was observed between mRNA variant expression and patient survival. However, *b2a2* was indicative of patients with longer survival as well as those treated with imatinib or nilotinib. Additionally, platelet count could be a marker of *BCR::ABL1* translocation.

## 1. Introduction

Chronic myeloid leukemia (CML) is a clonal hematological malignancy, meaning it originates from a single mutated cell in the bone marrow. This mutation disrupts normal blood cell production, leading to the uncontrolled growth of mature myeloid cells and their precursors, specifically those in the granulocyte lineage. This abnormal proliferation manifests clinically as an elevated white blood cell count, with these abnormal cells present in both the bone marrow and peripheral blood. Interestingly, most patients with CML are asymptomatic, particularly in the early stages. However, as the disease progresses, some individuals may develop a constellation of characteristic symptoms. These can include fatigue without an apparent cause, unexplained weight loss, and a pale appearance due to anemia. Additionally, some patients may experience skin problems, splenomegaly, or hepatomegaly. Furthermore, B symptoms, which include fever, night sweats, and unintentional weight loss, can also occur [1]. Notably, CML primarily strikes adults between the ages of 52 and 64, accounting for roughly 15–20% of all adult leukemias. This age group demonstrates a higher incidence compared to others, with men exhibiting a slightly greater susceptibility [2].

Importantly, CML has three phases: chronic [3], accelerated [4], and blast crisis, either myeloid or lymphoid crisis, which can progress to a fatal phase in approximately 5 years [5]. One essential feature of this neoplasia is the presence of the reciprocal translocation t(9;22)(q34;q11), which is found in approximately 90% of cases. This translocation gives rise to the Philadelphia chromosome (Ph), a fusion of *ABL* (9q34) and *BCR* (22q11) genes, creating a hybrid *BCR::ABL1* gene with a chimeric messenger RNA (mRNA), encoding for a protein with an exacerbated tyrosine kinase activity. This gene fusion is a key point for CML pathogenesis because the oncoprotein activates signaling pathways such as RAS/MEK, JAK/STAT, and PI3K/AKT promoting cell growth, cell survival, and inhibition of apoptosis [6]. Within the *BCR* gene, the most common sites for chromosomal breaks occur within a specific area called the major breakpoint cluster region (M-bcr). This region spans roughly 300 kb and is located between exons b2 and b3, or alternatively, between b3 and b4. Conversely, breakpoints within the *ABL* gene are typically found near the beginning (5′-region) of exon a2. These rearrangements in both genes lead to the formation of various fusion mRNA products. For instance, the *b2a2* mRNA variant (also known as e14a2) arises when exon b2 fuses with exon a2. Similarly, the *b3a2* mRNA variant (e13a2) forms when exon b3 fuses with a2 [6], predominantly with *b2a2* or *b3a2* junctions [7].

In this sense, less frequent rearrangements have also been documented, such as the *e1a2* (more frequent in Ph+ cases of Acute Lymphocytic Leukemia) or *b3a3* mRNA variants, both found it in about 1% of CML cases [8].

It has been reported that the frequency of the mRNA variants varies across different populations [9]. Although *b3a2* is the most frequently reported mRNA variant in Mexico [10], studies on the frequency and importance of *BCR::ABL1* mRNA variants in patients with CML are still limited. Therefore, this study aimed to investigate the distribution of *BCR::ABL1* mRNA variants in the different clinical phases of CML and their effect on hematological parameters, clinical variables, and patient survival in Mexican population.

## 2. Materials and Methods

The study examined 580 medical records from the hematology and oncology unit of the Bajio Regional High Specialty Hospital (Hospital Regional de Alta Especialidad del Bajio—HRAEB) in León Guanajuato, Mexico. These records belonged to patients diagnosed with leukemia between 2008 and 2018. Focusing on individuals from the Bajio region, the analysis included only male and female patients with Chronic Myeloid Leukemia (CML). All patients with immunophenotype and karyotype analysis, blood cell count, as well as *BCR::ABL1* mRNA variants genotyping via qPCR were selected. Patients with incomplete medical data (exceeding 70% missing information) or lacking a definitive diagnosis confirmed by either immunophenotyping or *BCR::ABL1* genotyping were excluded.

To analyze the data, the Shapiro–Wilk test assessed the distribution of quantitative variables. Inferential statistics were then performed using the Mann–Whitney U test for non-parametric comparisons and the chi-square test for categorical data analysis. A significance level of *p* < 0.05 was set, indicating a 95% confidence interval in all statistical tests. Kaplan–Meier curves evaluated patient survival rates, and Cox proportional hazards regression analyses were conducted to identify factors influencing survival over a 300-week follow-up period. GraphPad Prism software version 9.0.0 was used for all statistical analyses.

## 3. Results

### 3.1. Description of the Study Population

Of the 580 patients record, 51 had a CML diagnosis (8.8%), among which 18 were excluded owing to missing data, resulting in a total of 33 patients with complete medical records. Of these, 66.7% and 33.3% were male and female patients, respectively. In terms of clinical parameters, 9.1%, 21.2%, 57.6%, 30.3%, 18.2%, 3%, 6.1%, and 3% of patients had diabetes, hypertension, splenomegaly, hepatomegaly, anemia, hemorrhagic syndrome, thrombocytosis, and thrombocytopenia, respectively. Moreover, 3% of patients had adenomegaly or edema and ecchymosis or petechiae, respectively (Table 1).

### 3.2. Association between BCR::ABL1 mRNA Variants and Clinical Data

The patients were classified according to *BCR::ABL1* mRNA variant expression into “positive” (PP) (84.8%) and “negative” (NP) (15.2%). Patients in the PP group were diagnosed at an earlier age (median of 37.5 years) than those in the NP group (73 years) (*p* = 0.046) (Table 2).

In terms of the clinical phase of CML, 3.6%, 25%, and 71.4% of the PP group were in the accelerated, blast, and chronic phases, respectively, while 100% of the NP group were in the chronic phase. No significant association was found when comparing the frequency of the different clinical phases between groups (*p* = 0.38) (Table 2). However, significant differences were found when analyzing hematological parameters, with high platelet (*p* = 0.004) and basophil (*p* = 0.025) counts in the PP group, while parameters such as leukocyte, erythrocyte, neutrophil, eosinophil, and blast counts did not show significant differences (Table 2).

### 3.3. Association between mRNA Variants and the Clinical Phase of CML

The most frequently detected mRNA variant was *b3a2* (64.3%), followed by *b2a2* (28.6%), *e1a2* (3.6%), and *b3a2*/*b3a3* co-expression (3.6%) (Table 2).

The PP group (n = 28) was stratified by mRNA variant type as follows: *b3a2* (n = 18), *b2a2* (n = 8), and ‘Other mRNA variants’ (n = 2). In the *b2a2* and *b3a2* subgroups, patients were diagnosed in the accelerated and chronic phases at rates of 12.5% and 87.5%, and 27.8% and 72.2%, respectively. For the ‘Other mRNA variants’ subgroup, 100% of patients were in the blast phase. Inferential analysis revealed no significant association between the clinical phase at diagnosis and mRNA variant type (Table 3). Furthermore, in terms of mRNA variant expression and age at diagnosis, our analysis indicated that patients expressing *b2a2* are diagnosed at a later age compared to those expressing *b3a2* (*p* = 0.02) (Table 3). Subsequently, an evaluation of treatment types revealed that mRNA variant type does not serve as a determining factor when selecting either the initial (*p* = 0.40) or final (*p* > 0.99) treatment. Specifically, based on mRNA variant type alone, the treatment selection appears to be arbitrary (Table 4). Importantly, it should be noted that no significant variations were observed in hematological parameters (Table 3).

### 3.4. Association between the Clinical Phase of CML and Clinical Variables

The patients were classified into two groups: (1) chronic phase (CP) (n = 25; 75.8%); (2) advanced phase (AP) (n = 8; 24.2%) (this latter group consisted of accelerated and blast phases). In the AP group, 62.5% and 37.5% were male and female patients, respectively, while in the CP group, the percentages were 68% and 32% for men and women, respectively (Table 4). Furthermore, patients in the CP group had a higher median age, both at admission and at diagnosis, although the difference was not significant (Table 4).

An analysis of the type of treatment, both initial and final, showed no significant association.

With respect to hematological parameters, significant differences were only found for the erythrocyte counts (*p* = 0.01) and percentage of blasts (*p* = 0.09). Moreover, the CP group showed a higher erythrocyte count, while the AP group had a higher percentage of blasts (Table 4).

Regarding mRNA variant type, 52% of patients in the CP group expressed *b3a2*, 28% expressed *b2a2*, and 20% were negative. For the AP group, 62.5% and 12.5% of patients expressed *b3a2* and either *b2a2*, *b2a2*/*b3a3*, or *e1a2*, respectively. However, no significant association was found between mRNA variant type and clinical phase of CML (*p* = 0.07) (Table 4).

### 3.5. Survival Analysis

**Figure 1:**
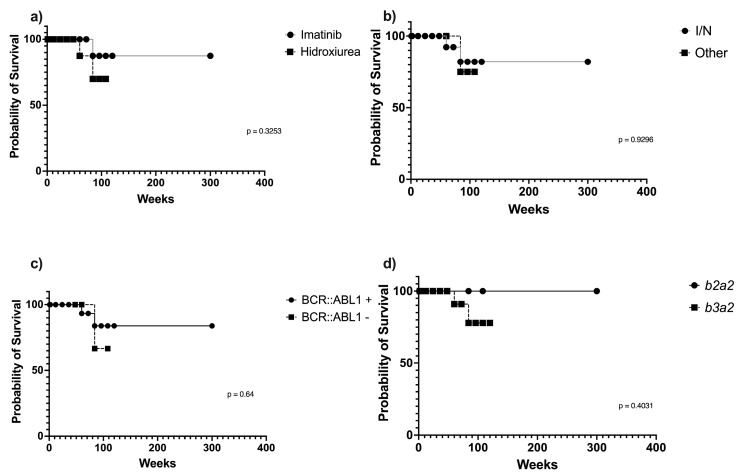
Kaplan–Meier survival curves. (**a**) Survival analysis based on the initial treatment (imatinib or hydroxyurea); no significant differences were found in patient survival probability (*p* = 0.3253). (**b**) Survival analysis based on the final treatment (imatinib/nilotinib or others); no significant differences found between the two treatments (*p* = 0.9296). (**c**) Survival analysis based on the presence of the *BCR::ABL1* translocation; no significant differences (*p* = 0.64) found between the two groups. (**d**) Survival probability of patients expressing *b2a2* or *b3a2* mRNA variants; no significant differences (*p* = 0.4031) found between the two groups. I/N = treatment with imatinib or nilotinib; Other = treatment with hydroxyurea, hydroxycarbamide, and allopurinol.

Since the type of treatment showed a random pattern, either in terms of the clinical phase of CML or the mRNA variant type, a survival analysis was performed based on the initial or final treatment, grouping the treatment regimens into imatinib or nilotinib (tyrosine kinase inhibitors) and hydroxyurea or others (hydroxycarbamide and allopurinol). Notably, no significant differences in survival probability were observed in both cases. However, a longer survival trend was found in patients treated with tyrosine kinase inhibitors, either as the initial (Figure 1a) or final (Figure 1b) treatment, but this finding should be further confirmed with a larger sample size.

Furthermore, Kaplan–Meier analyses were performed to determine whether patient survival was related to the presence of *BCR::ABL1* or to mRNA variant expression. No association was found between these parameters and survival probability, although a slightly longer survival trend was observed in the presence of the *BCR::ABL1* translocation (Figure 1c) and with the expression of the *b2a2* mRNA variant (Figure 1d).

## 4. Discussion

This study found a median age of 40 years at diagnosis with 66.7% of male patients, consistent with other study populations presenting a median age of 51.7 ± 12.2 years at diagnosis with a high prevalence of male cases (58%, on average) [11,12].

It is important to note that PP patients showed a median age of diagnosis at 37.5 years, which is younger compared to worldwide median (50 years); this could be attributed to occupational exposure to volatile organic compounds, such as benzene and aromatic compounds, frequently used in the fur industry, which is the most important commercial activity in the Bajio Region; nevertheless, this assumption does not have sufficient evidence, and more studies are needed in order to probe it [13,14,15].

On the other hand, our study revealed an interesting peculiarity of the Bajio population, and it was the high proportion of CML patients negative to *BCR::ABL1* transcript expression (15%), also called atypical CML (aCML). These findings are inconsistent with worldwide aCML prevalence (approximately 1%). In this sense, it is important to know that there is a lack of data for the risk factors classification of aCML; however, aging is considered an important risk factor, and it may be that the population aging process could be the reason for this high proportion of aCML, or it could be a region-specific phenomenon; nevertheless, these arguments need to be better explored, in order to elucidate if this observation is not a consequence of the limited sample studied in our work [16,17].

Regarding the clinical phase, the chronic phase was the most frequent (71.4%), followed by the blast (25%) and accelerated (3.6%) phases, consistent with reports from other countries showing a high frequency for the chronic phase (about 50%), followed by the blast phase and the accelerated phase [18,19]. Nevertheless, some differences may be attributed to the heterogeneity and scope of the studies.

Concerning hematological parameters, several studies have reported increased leukocyte and platelet counts, with medians of 191.6 × 10^9^ and 462.0 × 10^3^ cells/L, respectively [20]. Although this study found no significant differences for leukocyte counts, increased platelet counts were associated with the expression of specific *BCR::ABL1* mRNA variants types, with a median of 465 × 10^3^ cells/L, consistent with previous reports.

As for blasts, these were detected in only 42% (14/33) of patients, with a median of 5%. However, several studies have reported substantial differences for this cell type, ranging from 1% to 5%, suggesting a highly variable behavior in the population of these cells [20].

Our study found that *b3a2* (64.3%) was the most frequent mRNA variant, followed by *b2a2* (28.6%), *e1a2* (3.6%), and *b3a2*/*b3a3* co-expression (3.6%). In this context, *b3a2* is the most prevalent mRNA variant in various Western populations [21,22], whereas in Bolivia, *b2a2* is the most common mRNA variant, followed by *b3a2* [23]. In Mexico, *b3a2* predominates, succeeded by *b2a2* [10,24]. However, a multicenter study carried out in Mexico (1997–2001) reported *b2a2* as the most frequent mRNA variant [25], with other South American countries showing the same trend [26]. Such variability may be due to factors related to the study methodology, sample size and/or genetic ancestral contribution of the population, suggesting that ancestry should be considered a key factor in the epidemiological description of the mRNA variants expressed in CML.

With respect to the co-expression phenomenon, it has been suggested that it may be due to alternative splicing or cellular heterogeneity resulting in different *BCR::ABL1* gene expression profiles [27].

Comparing the clinical variables by mRNA variant type showed no significant differences in the hematological parameters. However, higher leukocyte, platelet, and eosinophil counts are apparently found, at least in *b3a2*-positive cases, while a non-significant increase in neutrophil counts was observed in *b2a2*-positive cases. This is consistent with other reports [22] and may be due to the interaction of *BCR::ABL1* products, which can affect the cytoplasmic fragmentation of megakaryocytes, platelet formation, cytoskeletal changes, and cell adhesion and motility [28].

As for the clinical phase of CML, the chronic phase is the first stage of the disease, and it is well known that about 80–90% of patients Ph+ in this phase, as found in the present study, with 80% of patients testing positive for the presence of a *BCR::ABL1* mRNA variant [29].

Survival analysis according to mRNA variant type showed no significant differences in survival probability in the study population. However, it is important to note that *b2a2*-positive patients had 100% survival up during 300 weeks of follow-up, while *b3a2*-positive patients had only 75% survival at 120 weeks, which differs from the findings of other studies that have reported that *b3a2*-positive patients have higher survival rates compared to *b2a2*-positive patients, at least for a follow-up up to five years [18,19]. Nevertheless, the observations in the current study may be attributed to the clinical phase at diagnosis. Specifically, cases positive for *b3a2* were predominantly diagnosed in the chronic (72.2%) and blast (27.8%) phases, while those positive for *b2a2* were chiefly diagnosed in the chronic phase (87.5%), with a minimum of 12.5% in the accelerated phase. Furthermore, the transition to the accelerated or blast phase is usually associated with additional chromosomal abnormalities and probably with mutations in other proto-oncogenes [30], potentially impacting the duration of the clinical phases and, therefore, patient survival [31].

According to the survival outcomes of the final treatment, the long-term treatment with tyrosine kinase inhibitors (imatinib and nilotinib) has shown higher survival rates than hydroxyurea or other treatments [18]. Specifically, patients in the chronic phase who were treated with imatinib advanced to the accelerated phase within eight months, whereas those treated with hydroxyurea progressed in a mere three months. Consequently, it can be inferred that patients receiving treatment without tyrosine kinase inhibitors are more prone to advance to the accelerated phase of CML, ultimately leading to a blast crisis, which may be myeloid or lymphoid. In this regard, treatment with imatinib has been reported to be more efficient in patients with CML in the chronic phase, although the risk of disease progression still exists [32].

Our study is limited by a small sample size. As the Bajio Regional High Specialty Hospital is not primarily focused on treating patients with CML, recruitment of a larger patient sample was not achieved, even though it was conducted from 2008 to 2018. Nonetheless, several of the study findings have been reported in other populations. Moreover, this study analyzed treatment-associated survival, which has been poorly investigated.

## 5. Conclusions

The data obtained in this study suggest that the Mexican population of Bajio has a higher frequency of the *b3a2* mRNA variant. Moreover, *b3a2* was more frequent in the chronic phase of CML. No significant differences were found in survival; nevertheless, a tendency for longer survival trend was observed in patients expressing *b2a2* and those treated with tyrosine kinase inhibitors. All these observations must be better explored in a bigger study cohort. It is important to note the association of the presence of the *BCR::ABL1* translocation with an increased platelet count, suggesting this parameter is a possible indicator of translocation. In addition, considering the clinical phase, patients in the chronic phase showed an increased erythrocyte count. On the other hand, it is important to consider that one of the main limitations of this study is that the results obtained are from a single center, and the obtained results should be explored and validated in a larger, multicenter population of the region.

## Figures and Tables

**Table 1:** Description of clinical data of patients with CML.

Total (n = 33)	
Diabetes	
Yes (%)	3 (9.1%)
No (%)	30 (90.9%)
Hypertension	
Yes (%)	7 (21.2%)
No (%)	26 (78.8%)
Splenomegaly	
Yes (%)	19 (57.6%)
No (%)	14 (42.4%)
Hepatomegaly	
Yes (%)	10 (30.3%)
No (%)	23 (69.7%)
Anemia	
Yes (%)	6 (18.2%)
No (%)	27 (81.8%)
Hemorrhagic syndrome	
Yes (%)	1 (3%)
No (%)	32 (97%)
Thrombocytosis	
Yes (%)	2 (6.1%)
No (%)	31 (93.9%)
Thrombocytopenia	
Yes (%)	1 (3%)
No (%)	32 (97%)
Adenomegaly/Edema	
Yes (%)	1 (3%)
No (%)	32 (97%)
Ecchymosis/Petechiae	
Yes (%)	1 (3%)
No (%)	32 (97%)

**Table 2:** Description of demographic and clinical data of patients with CML stratified by *BCR::ABL1* mRNA variants positivity.

	PP (n = 28)	NP (n = 5)	*p*
Demographic data			
Female (%)	9 (32.1)	2 (40.0)	>0.99
Male (%)	19 (67.9)	3 (60.0)	
Age at diagnosis	37.5 (20.8–49.8)	73.0 (35.5–79.5)	0.046
Age at admission	42.5 (28.3–54.0)	82.0 (41.0–85)	0.037
Clinical phase of CML at diagnosis			
Accelerated (%)	1 (3.6)	0 (0)	0.38
Blast (%)	7 (25)	0 (0)	
Chronic (%)	20 (71.4)	5 (100.0)	
Hematological parameters			
Leukocytes (10^9^ cells/L)	175.3 (69.4–281.2)	101.4 (34.45–292.6)	0.33
Erythrocytes (10^9^ cells/L)	2.9 (2.6–3.9)	3.3 (2.5–3.8)	0.89
Platelets (10^9^ cells/L)	465.0 (287.8–599.0)	121.0 (68–133.5)	0.004
Neutrophils (%)	73.0 (61.0–81.0)	71.0 (47.0–77.0)	0.50
Basophils (%)	6.0 (2.3–9.8)	0.0 (0.0–3.5)	0.025
Eosinophils (%)	2.0 (1.0–5.8)	2.0 (0–10.5)	0.94
Blasts (%) *	0.5 (0.0–5.5)	0	0.08
mRNA variants			
*b2a2*	8 (28.6%)	0 (0%)	
*b3a2*	18 (64.3%)	0 (0%)	
*e1a2*	1 (3.6%)	0 (0%)	
*b3a2/b3a3*	1 (3.6%)	0 (0%)	
Negative	0 (0%)	5 (100.0%)	

PP: patients with *BCR::ABL1* mRNA variant expression; NP: patients without *BCR::ABL1* mRNA variant expression; CML: chronic myeloid leukemia. * The analysis of the % of blasts only included patients having this type of cell.

**Table 3:** Description of demographic and clinical data of patients with CML stratified by type of *BCR::ABL1* mRNA variants.

	*b2a2* (n = 8)	*b3a2* (n = 18)	Other (n = 2)	*p*
Female (%)	3(37.5)	5 (27.8)	1 (50.0)	0.66
Male (%)	5 (62.5)	13 (72.2)	1 (50.0)	
Age at diagnosis	50.0 (33.3–55.8)	32.5 (17.3–45.0)	31.5 (14.0–49.0)	0.02
Clinical phase of CML at diagnosis				
Accelerated (%)	1 (12.5)	0 (0)	0 (0)	0.09
Blast (%)	0 (0)	5 (27.8)	2 (100.0)	
Chronic (%)	7 (87.5)	13 (72.2)	0 (0.0)	
Type of treatment (initial)				
Imatinib (%)	3 (37.5)	11 (61.1)	1 (100.0)	0.40
Hydroxyurea (%)	5 (62.5)	7 (38.9)	0 (0.0)	
Type of treatment (final)				
I/N (%)	7 (87.5)	15 (83.3)	2 (100)	>0.99
Other (%)	1 (12.5)	3 (16.7)	0 (0)	
Hematological parameters				
Leukocytes (10^9^ cells/L)	159.1 (46.11–298.9)	208.2 (132.1–292.1)	0.5 (0.2–0.8)	0.46
Erythrocytes (10^9^ cells/L)	3.49 (2.7–4.4)	2.9 (2.6–3.5)	2.3 (2.2–2.5)	0.19
Platelets (10^9^ cells/L)	437.5 (318.0–599.0)	500.5 (299.0–745.8)	8.5 (0.0–17.0)	0.80
Neutrophils (%)	79.5 (68.3–83.3)	73.0 (56.0–82.0)	7.0 (0.0–14.0)	0.39
Basophils (%)	6.0 (3.5–10.0)	6.5 (3.5–10.0)	1.0 (0.0–2.0)	0.90
Eosinophils (%)	2.0 (1.3–5.3)	3.0 (0.8–6.2)	0.5 (0.0–1.0)	0.97
Blasts (%) *	3.0 (2.0–4.0)	12.0 (2.0–20.0)	0.0	0.23

CML: chronic myeloid leukemia, I/N: treatment with imatinib or nilotinib. * The analysis of the % of blasts only included patients having this type of cell. The *p*-value corresponds to the comparison *b2a2* vs. *b3a2*.

**Table 4:** Description of demographic and clinical data of patients with CML stratified by clinical phase.

	CP (n = 25, 75.8%)	AP (n = 8, 24.2%)	*p*
Female (%)	8 (32)	3 (37.5)	>0.99
Male (%)	17 (68)	5 (62.5)	
Age at diagnosis	40.0 (26.5–59.5)	37.5 (18.2–48.0)	0.47
Age at admission	49.0 (30.0–63.5)	42.5 (25.8–53.0)	0.46
Type of treatment (initial)			
Imatinib (%)	14 (56.0)	4 (50.0)	0.77
Hydroxyurea (%)	10 (40.0)	4 (50.0)	
No treatment (%)	1 (4.0)	0 (0)	
Type of treatment (final)			
I/N (%)	20 (80.0)	7 (87.5)	0.81
Other (%)	4 (16.0)	1 (12.5)	
No treatment (%)	1 (4.0)	0 (0)	
Status			
Deceased (%)	1 (4.0)	2 (25.0)	0.13
Alive (%)	24 (96.0)	6 (75.0)	
Hematological parameters			
Leukocytes (10^9^ cells/L)	172.4 (62.5–280.1)	140.3 (27.2–291.4)	0.57
Erythrocytes (10^9^ cells/L)	3.2 (2.8–3.9)	2.6 (2.3–2.8)	0.01
Platelets (10^9^ cells/L)	414.0 (150.5–589.5)	406.0 (11.8–581.8)	0.52
Neutrophils (%)	72.5 (65.8–80.3)	62.0 (20.5–86.3)	0.69
Basophils (%)	5.0 (0.0–8.0)	7.5 (0.5–12.8)	0.44
Eosinophils (%)	2.0 (1.0–6.0)	1.0 (0.0–3.5)	0.16
Blasts (%) *	4.0 (2.0–12.0)	44.0 (4.0–79.0)	0.09
*BCR::ABL1*			
Positive (%)	20 (80.0)	8 (100.0)	0.30
Negative (%)	5 (20.0)	0 (0)	
Types of mRNA variants			
*b2a2*	7 (28.0)	1 (12.5)	0.07
*b2a2*/*b3a3*	0 (0)	1 (12.5)	
*b3a2*	13 (52.0)	5 (62.5)	
*e1a2*	0 (0)	1 (12.5)	
Negative	5 (20.0)	0 (0)	

* The analysis of the % of blasts only included patients having this type of cell. CP: chronic phase; AP: advanced phase; I/N: treatment with imatinib or nilotinib.

## Data Availability

The data that support the findings of this study are available on request from the corresponding author. The data are not publicly available due to the data also forms part of an ongoing study.
